# Comparison of real-time PCR and nested PCR for toxoplasmosis diagnosis in toxoplasmic retinochoroiditis patients

**DOI:** 10.1186/s12879-021-06873-3

**Published:** 2021-11-23

**Authors:** Khadijeh Khanaliha, Farah Bokharaei-Salim, Alireza Hedayatfar, Abdoulreza Esteghamati, Sayyed Amirpooya Alemzadeh, Qasem Asgari, Saba Garshasbi, Borna Salemi

**Affiliations:** 1 Research Center of Pediatric Infectious Diseases, Institute of Immunology and Infectious Diseases, Iran University of Medical Sciences, Tehran, Iran; 2 Department of Virology, School of Medicine, Iran University of Medical Sciences, Tehran, Iran; 3 Eye Research Center, The Five Senses Institute, Iran University of Medical Sciences, Tehran, Iran; 4 Noor Ophthalmology Research Center, Noor Eye Hospital, Tehran, Iran; 5 Department of Parasitology and Mycology, School of Medicine, Shiraz University of Medical Sciences, Shiraz, Iran; 6 Iran University of Medical Sciences, Tehran, Iran; 7 Student Research Committee, School of Medicine, Iran University of Medical Sciences, Tehran, Iran

**Keywords:** *Toxoplasma gondii*, Retinochoroiditis, Real-time PCR, Ocular toxoplasmosis, PCR, Peripheral blood mononuclear cells

## Abstract

**Backgrounds:**

PCR is a proper technique that significantly improves toxoplasmosis diagnosis. However, a more sensitive technique is required. This study compared real-time PCR with nested PCR using B1, SAG-4, and MAG-1 bradyzoite genes to diagnose toxoplasmosis in toxoplasmic retinochoroiditis patients.

**Methods:**

Blood samples were collected from 10 patients with active toxoplasmic chorioretinal lesions and 10 healthy individuals. Blood samples including peripheral blood mononuclear cells (PBMCs), serum and whole blood samples were used for DNA extraction. Serum was also used to detect anti-toxoplasma IgG and IgM antibodies. Nested PCR and real-time PCR were performed using B1, SAG-4, and MAG-1 target genes.

**Results:**

Five (50%) out of the 10 patients were tested positive for toxoplasmosis with nested PCR using the PBMC samples. All the five patients tested positive with nested PCR were also tested positive for toxoplasmosis with real-time PCR using the PBMC samples. The real-time PCR results demonstrated that 9(90%) out of the 10 patients were positive based on B1 and the remaining one (10%) was positive only based on MAG-1. In general, of the patients, five (50%) were positive using SAG-4 and three (30%) were positive in term of MAG-1 using PBMCs with real-time PCR.

**Conclusion:**

It appears that PBMC samples have the best performance as the PCR extraction method and are a good source for toxoplasmosis diagnosis. The use of B22 and B23 target genes due to their high sensitivity and specificity along with bradyzoite genes are recommended for toxoplasmosis diagnosis using PBMC samples with real-time PCR.

## Background

*Toxoplasma gondii* protozoan parasite is an important cause of retinochoroiditis throughout the world. Toxoplasmic retinochoroiditis caused by acute infection or recurrence is often found in congenital or acquired toxoplasmosis [1].This disease typically affects at the posterior pole of a single eye, with solitary, multiple, or satellite lesions to a pigmented retinal scar [2]. Active lesions present as grey-white focuses of retinal necrosis with adjacent choroiditis, vasculitis, hemorrhage, and vitritis [2].

Toxoplasmosis is as one of the most common parasitic infections in the world and has been reported in up to one third of the world’s population.It is responsible for the majority of uveitis or intraocular inflammation cases. In some countries, up to 50% of all posterior uveitis cases are attributed to toxoplasmosis [3–5].

The polymerase chain reaction (PCR) technique has obviously improved toxoplasmosis diagnosis and is a proper technique for parasite detection in clinical samples. Although PCR is a useful technique for detection of *T. gondii* DNA with sensitivity between 53.3% -75% in blood samples of ocular toxoplasmosis patients, a more sensitive technique is still required [6–8].

Recently, the real-time PCR technique as a highly secure and efficient method has been extensively used for toxoplasmosis diagnosis using different target genes [9–11]. It has been replacing nested PCR as a sensitive technique for rapid ocular toxoplasmosis diagnosis. As described previously, this method decreases contamination risks, confirms specificity, and omits some gel production processes in the conventional PCR [12]. Furthermore, in this method, standardization improves and the parasitic load is quantified in samples for screening test and treatment evaluation [13].

The results of some previous studies indicated that *T. gondii* bradyzoite (SAG-4 and MAG-1) genes are useful targets for diagnosis of toxoplasmosis in human immunodeficiency virus (HIV) positive and toxoplasmic retinochoroiditis patients, especially when patients were under treatment or prophylaxis. Moreover, PCR using *T. gondii* B1 gene showed good performance for diagnosis of toxoplasmosis [14–16].

In this study, we compared the performances of real-time and nested PCR using B1, SAG-4, and MAG-1 bradyzoite genes for toxoplasmosis diagnosis in the peripheral blood mononuclear cell (PBMC) samples of patients with toxoplasmic retinochoroiditis.

## Methods

Blood samples were collected from 10 patients with toxoplasmic chorioretinal lesions admitted to ophthalmology clinic in the Rasoul Akram Hospital in Tehran, affiliated to Iran University of Medical Sciences during 2019–2020 and from 10 healthy individuals. The mean age of the patients with an active retinitis lesion was 24.5 ± 6.19 years (ranging from 12 to 42 years) and for healthy people was 28.6 ± 8.3 (ranging between 15 and 45 years). Among ocular patients and healthy controls, 6/10 (60%) and 5/10 (50%) were male, respectively. In this study, we included a total of 10 toxoplasmic retinochoroiditis outpatients from different provinces of Iran, including Mazandaran (Sari, Amol), Golestan (Gorgan), Khuzestan (Ahvaz), Markazi (Arak), Qom, and Tehran. The most toxoplasmic chorioretinal lesions were seen in the right eye and less in the left eye.

All the ocular patients had an active retinitis lesion adjacent to an old hyperpigmented retinal scar compatible with ocular toxoplasmosis reactivation. Clinically detectable retinal vasculitis was recorded in seven of the patients. The patients had moderate to severe vitritis at the time of diagnosis.

Blood samples including PBMC and serum as well as whole blood samples were used to extract DNA and also serum was used to detect anti-*Toxoplasma* IgG and IgM antibodies. PBMC was prepared from 5 mL blood with Ethylenediamine tetraacetic acid (EDTA) through density gradient centrifugation using Ficoll (Amersham Biosciences Europe Gmbh, Milan, Italy) [16].

### ELISA: Enzyme-linked immunosorbent assay

The anti-*T.gondii* IgG and IgM antibodies were evaluated using Enzyme Linked Immunosorbent Assay (Euroimmun, Germany) for all the 10 ocular toxoplasmosis patients and 10 healthy individuals. Moreover, the presence or absence of the anti-*T.gondii* IgG and IgM antibodies was evaluated. According to results positive and negative IgG were considered as > 11 IU/mL and < 8 IU/mL, respectively, positive and negative IgM were defined as > 1.1 IU/mL and < 0.8 IU/mL, respectively.

### DNA extraction and polymerase chain reaction

The genomic DNA was extracted from the patients’ serum and also PBMC and whole blood samples were obtained using the QIAamp DNA mini kit (Qiagen, Hilden, Germany). The primers were selected using B1 that amplified a 115 bp fragment [17]. Nested PCR was performed using SAG-4 primers that amplified a 187 bp fragment, as previously described in detail [15, 18]. The oligonucleotide was used for MAG-1 that amplified a 212 bp fragment [15]. All the PCR protocols and conditions were performed according to Contini et al.’s method [14, 15].

### Sequencing

The next round of PCR was performed with internal primers, and the product was purified using the High Pure PCR Product Purification Kit (Roche Diagnostic, Mannheim, Germany). Then, the product was used for direct sequencing with the dye termination method and an ABI 3730xl sequencer. The DNA sequence was analyzed with genius software version 10.1. Finally, the results were compared and new sequences were deposited with the accession numbers (MZ027341 to MZ027344 and also MZ054158 to MZ054160) in GenBank.

### Real-time polymerase chain reaction

The real-time PCR test was performed using *T.gondii* B1, SAG-4, and MAG-1 genes. The reactions were performed in a final volume of 20 µl using the Master SYBR Green I (Roche Molecular Biochemicals) 12.5 µl. Each primer was added at the concentrations of 0.5 µM for B1, 0.8 µM for SAG-4, 0.7 µM for MAG-1 and MgCl_2_, 2 mM for B1, 4 mM for SAG-4 and MAG- 1 and then 5 µl of the extracted DNA samples [14].

All runs were performed with distilled water as negative control and a DNA sample of *T.gondii* RH strain as positive control. The test specificity was assessed using B1, SAG-4, and MAG- 1 primers through DNA extraction from the *T.gondii* RH strain and DNA preparation from *T. gondii* positive patients and from10 healthy individuals as control. Some bacterial species (*Staphylococcus aureus*, *Streptococcus pneumoniae* and *Pseudomonas aeruginosa*) and fungal species (*Candida* *albicans*, *Candida glabrata* and *Cryptococcus neoformans*) and viruses, including cytomegalovirus (CMV), herpes simplex virus were used in this study. Primers used in this study for real-time PCR are summarized in Table 1.

**Table.1:** Primers used in nested and real-time PCR

Primers		Sequences	Annealing	Amplified fragment (bp)	Refs
Forward	B1–B22	5′-AACGGGCGAGTAGCACCTGAGGAGA-3′	62	115	Bretagne,1993 [17]
Reverse	B1–B23	5′-TGGGTCTACGTCGATGGCATGACAAC-3′			
Forward	SAG-4-OP1	5′-TGGACCTACGATTTCAAGAAGGC-3′	60	187	Odberg-Ferragut,1996 [18]
Reverse	SAG-4-OP2	5′-GCTGCGAGCTCGACGGGCTCATC-3′			
Forward	MAG-1-IP1	5′-GCATCAGCATGAGACAGAAGA-3′	53	212	Contini, 2002 [15]
Reverse	MAG-1-IP2	5′-CCAACTTCGAAACTGATGTCG			

### Statistical analysis

Statistical analysis was done in SPSS version 18, using Chi-square test. Predictive tests were used to analyze and compare the results of Real-Time PCR and nested PCR using different primers (SAG-4, MAG-1, and B1) in the PBMCs and serum samples of the patients. The difference was significant at P < 0.05.

## Results

### PCR result

Five (50%) out of the 10 patients with toxoplasmic retinochoroiditis were positive with nested PCR using the PBMC samples. In general four (40%) of patients were positive results using MAG-1 or SAG-4 and one (10%) patient was positive using B1 with nested PCR. Two of the patients were positive regarding MAG-1 and SAG-4. Moreover one patient was positive with MAG-1 and another was positive using SAG-4 gene. The PCR results were negative in patients’ serum samples using different targets (Table 2).

**Table 2:** Comparison of Real-time PCR and Nested PCR methods in diagnosing of toxoplasmosis in retinochoroiditis patients

Genes	MAG-1						SAG-4							B1						
No	Real-time PCR			PCR			Real-time PCR				PCR			Real-time PCR				PCR		
Samples	P	S	B	P	S	B	P		S	B	P	S	B	P	S	B		P	S	B
1	** + **	**−**	**−**	** + **	**−**	**−**	**−**		**−**	**−**	**−**	**−**	**−**	**−**	**−**	**−**		**−**	**−**	**−**
2	**−**	**−**	**−**	**−**	**−**	**−**	**−**		**−**	**−**	**−**	**−**	**−**	** + **	**−**	**−**		**−**	**−**	**−**
3	**−**	**−**	**−**	**−**	**−**	**−**	**−**		**−**	**−**	**−**	**−**	**−**	** + **	**−**	**−**		**−**	**−**	**−**
4	**−**	**−**	**−**	**−**	**−**	**−**	**−**			**−**	**−**	**−**	**−**	** + **	**−**	**−**		**−**	**−**	**−**
5	**−**	**−**	**−**	**−**	**−**	**−**	**−**		**−**	**−**	**−**	**−**	**−**	** + **	**−**	**−**		** + **	**−**	**−**
6	** + **	**−**	* +	** + **	**−**	**−**	** + **		**−**	** + **	** + **	**−**	**−**	** + **	**−**	** + **		**−**	**−**	**−**
7	**−**	**−**	**−**	**−**	**−**	**−**	** + **		**−**	**−**	** + **	**−**	**−**	** + **	**−**	**−**		**−**	**−**	**−**
8	**−**	**−**	**−**	**−**	**−**	**−**	** + **		**−**	**−**	**−**	**−**	**−**	** + **	**−**	**−**		**−**	**−**	**−**
9	**−**	**−**	**−**	**−**	**−**	**−**	** + **		**−**	**−**	**−**	**−**	**−**	** + **	**−**	**−**		**−**	**−**	**−**
10	** + **	**−**	** + **	** + **	**−**	**−**	** + **		**−**	** + **	** + **	**−**	**−**	** + **	**−**	** + **		**−**	**−**	**−**
Total	3	0	2	3	0	0	5		0	2	3	0	0	9	0	2		1	0	0

P = peripheral blood mononuclear cells (PBMCs), S = Serum, B = Blood, PCR: Polymerase Chain Reaction

*In blood samples that nested PCR were negative real-time were positive

The real-time PCR results demonstrated that all the 10 patients with toxoplasmic retinochoroiditis were positive. Accordingly, nine (90%) out of the 10 patients were positive with B1 and the remaining one (10%) was positive only with MAG-1 using the PBMC samples. In general, five (50%) of the patients were positive using SAG-4 (including two patients with SAG-4 and MAG-1) and three (30%) of the patients were positive using MAG-1.

All the five positive patients with nested PCR were also positive with real-time PCR using the PBMC samples. In two of the patients with negative PCR results using MAG-1, SAG-4, and B1 with nested PCR, the real-time PCR results were positive using SAG-4.

Furthermore, in two of the patients, the PCR results were negative in the blood samples of patients with MAG-1, SAG-4, with nested PCR. However, the PCR results were positive in the blood samples of these patients with real-time PCR. In general, nine (90%) of the cases were positive with real-time PCR in PBMC samples in compare with two (20%) were positive in blood samples using B1 gene.The results of real-time PCR and nested PCR are summarized in Table 2.

There was a significant difference between real-time PCR and nested PCR using B1 gene (90% vs. 10%; P = 0.0003). Moreover the use of bradyzoite-specific genes (SAG-4) was found to be more sensitive in real-time PCR than nested PCR (50% vs. 30%. P = 0.36) but the difference was not significant. Furthermore no significant difference was found in detecting MAG-1 between real-time PCR and nested PCR (30% vs. 30%; P = 0.50) in patients with toxoplasmic chorioretinitis.

### Real-time PCR specificity

The real-time PCR assays were performed using all target genes (MAG-1, SAG-4, and B1) in a group of 10 healthy individuals and different fungal, bacterial and viral infections, for confirmation of specificity and results of real-time PCR were negative.

### Sequencing

The DNA sequence was analyzed, and the results were compared and blasted with deposited sequences in PubMed. The B1 obtained sequences had 100% identity with the *T.gondii* B1 gene, the RH strain (AF179871.1), 98.63% identity with *T. gondii* ME49 (XM_002370240.2) and 98.26% identity with TPA: *T.gondii* VEG (LN714499.1).

One isolate of MAG-1 had 100% similarity with U09029 and *T.gondii* ME49 MAG1 (XM_002365659.1), and another isolate had 99.53% similarity with LN714498.1.Three isolates of SAG-4 had similarity with the *T.gondii* strain RH bradyzoite surface antigen (SAG-4) gene (genotype I).

### ELISA

Although anti-*T.gondii* IgG was positive in all the 10 retinochoroiditis patients, IgM was negative in them. Anti-*T.gondii* IgG was positive in 6 out of 10 healthy individuals; however IgM was negative in all of them.

## Discussion

Ocular toxoplasmosis is the most common cause of posterior uveitis, causing blindness [19]. It is diagnosed by observing necrotizing lesions on the ocular fundus, performing serological tests, and responding to treatment. The PCR technique has advantages for toxoplasmosis diagnosis in comparison with conventional methods [14]. Recently, real-time PCR has been used as a sensitive method for ocular toxoplasmosis diagnosis instead of nested PCR [20, 21].

In the present study, 10 patients with toxoplasmic retinochoroiditis and moderate to severe vitritis were evaluated using nested PCR and real-time PCR. According to the results, five (50%) patients who were positive using B1, SAG-4, and MAG-1 with nested PCR were also positive using real-time PCR and PBMC samples. Moreover, nine (90%) of the cases that were negative with nested PCR were positive using B1 and real-time PCR in the PBMC samples. Furthermore, two of the patients that were negative using SAG-4 with nested PCR were also positive with real-time PCR using the PBMC samples. The results of both nested PCR and real-time PCR were negative with these three target gens using serum samples in all the patients.

According to the results of present study, nine (90%) out of the 10 patients with retinochoroiditis were positive using B1 with real-time PCR. However, only one case was positive using B1 with nested PCR. This indicated good sensitivity for B1 (P22 and P23) for toxoplasmosis diagnosis using real-time PCR. Furthermore the real-time PCR (50%) was more sensitive than nested PCR (40%) for detection of SAG-4 /MAG-1 bradyzoite genes. These finding are in agreement with the results was reported in a study for diagnosis of ocular toxoplasmosis [14].

Contini et al., used bradyzoite-specific genes, along with B1 gene for diagnosis of toxoplasmosis in blood of patients with retinochoroiditis and result indicated that, the use of real-time PCR (56%) was more sensitive than nested PCR(19%) for detection of B1 gene and also real-time PCR using SAG-4/MAG-1 was more sensitive (59%) than nested PCR(39%) [14].

Our results support the idea that real-time PCR using B1 (B22 and B23) primers is more sensitive than nested PCR for diagnosing of toxoplasmosis.In some previous studies, the use of B22 and B23 primers was recommended for toxoplasmosis diagnosis due to their high sensitivity and specificity and good performance [12, 14]. It has been reported that B22 and B23 primers are highly sensitive in detecting 10^2^ to 10 ^−3^ T*.gondii*/ml using real-time PCR. However, the sensitivity limit of SAG-4 and MAG-1 was about 1 parasite/ml and thus B1 had a higher sensitivity than SAG-4 and MAG-1 genes for toxoplasmosis diagnosis [14].

Real-time PCR can contribute to toxoplasmosis diagnosis using blood samples and aqueous humor in ocular toxoplasmosis patients [22, 23]. It has been reported that sensitivities of real-time PCR assays using B1 gene is between 10 to 0.75 parasite genome equivalent per reaction [11, 12, 24].

The results of a study indicated that real-time PCR using B1 could detect *T.gondii* DNA in the aqueous humor samples of 16 (37.21%) patients, the peripheral blood samples of one (2.33%) patient, and both the blood and aqueous humor samples of seven (16.27%) patients among 43 patients with toxoplasmic retinochoroiditis [23].

In a study, 144 clinical specimens were evaluated with PCR technique using B1 and TaqMan-AF-PCR using the repetitive 529-bp sequence. The results indicated that all 72 cases were positive according to B1-PCR and Taq Man-PCR whereas 15 (20.8%) which were negative according to B1-PCR were positive according to TaqMan-AF-PCR. Generally, these results demonstrate the high sensitivity and specificity of the Taq Man-based PCR assay using a repeated sequence for toxoplasmosis diagnosis [25].

In a study, 30 fetal tissue sections were evaluated with nested and real-time PCR using B1. The results indicated that 10 (33%) sections were positive also, the outcomes obtained with real-time PCR were compatible with those obtained through nested PCR. The results also demonstrated that real-time PCR using B1 was highly sensitive and reproducible [26].

In another study, 46 biological samples were evaluated by nested PCR using B1 gene and also by two real- time PCR assay, Fluorescence Resonance Energy Transfer (FRET) and TaqMan protocols targeting a 529 bp repeat region and the 18S RNA gene, respectively. Three out of 46 samples were positive using nested PCR and these results were also confirmed by both real-time PCRs and result of study indicated that real-time PCR assays and nested PCR were sensitive and specific; however real-time PCR using FRET technology was more sensitive than TaqMan PCR and nested PCR [20].

In the present study, two of the cases were positive according to real-time PCR with the blood samples. However, the results were negative with nested PCR.So; another advantage of real-time PCR was positive PCR result using blood samples in comparison with nested PCR that had negative PCR result with blood samples.

Moreover, in the current study, 5 ml of whole blood was used to prepare PBMC samples that were finally used for DNA extraction. However, 200 µl of whole blood or serum samples were used for PCR extraction. The results indicated that PBMC was more efficient than blood or serum samples for DNA extraction. The results also demonstrated that PBMC could truly improve the molecular method for toxoplasmosis diagnosis, which is in line with some previous studies.

PBMC samples have been used to improve PCR diagnostic method in some previous studies, and they are among the good sources for *T. gondii* diagnosis, according to the previous research [16, 27].

In the present study, a blood sample was collected from one of the patients before and after specific treatment. The result indicated that after specific treatment; DNA was detected using B1 in the PBMC samples with real-time PCR. However, the copy number decreased with regard to anti-*Toxoplasma* treatment. The result of present study indicated the real-time PCR test can be useful for the monitoring of patients after treatment. This result was compatible with the results of Contini et al*.* (2005) and Costa et al*.* (2000), proposing that after treatment, DNA was still detectable in the blood and the copy number decreased depending on the application of anti-*Toxoplasma* treatment [14, 24].

In the present study, the MAG-1 obtained sequences from ocular toxoplasmosis patients had 100% identity with MAG-1 gene of the *T. gondii* ME49, that was reported by Parmley et al*.*, who previously sequenced the MAG-1 gene (GenBank- U09029) [28]. Furthermore, the B1 obtained sequence had 100% identity with *T. gondii* B1 gene, the RH strain (AF179871.1), that was previously described [29, 30].

Moreover, the PCR product sequencing of three SAG-4 positive cases with nested PCR demonstrated similarity with the *T. gondii* strain RH bradyzoite surface antigen (SAG-4) gene (genotype I). The PCR results using SAG2 in a study in Brazil demonstrated that type I *T. gondii* was the predominant strain in patients with ocular toxoplasmosis [31]. It has been proposed that type II *T. gondii* may be responsible for most cases of acquired ocular lesions, whereas type I *T. gondii* may be found more in congenital toxoplasmosis [2].

In general, real-time PCR is a simple and fast method that can be easily reproduced with low risks of contamination compared to the conventional PCR. Furthermore, real-time PCR with B22 and B23 along with SAG-4 and MAG-1 bradyzoite genes using PBMC samples is recommended for toxoplasmosis diagnosis and monitoring of patients after treatment. Also, the PBMC specimen is a proper supply for DNA extraction and can truly improve toxoplasmosis diagnosis.

Considering the low number of ocular toxoplasmosis cases in this study, future studies with large number of toxoplasmosis patients are recommended.

## Conclusion

PBMC samples had the best performance for amplification with the PCR method and were a good source for toxoplasmosis diagnosis. The use of B22 and B23 target genes due to their high sensitivity and specificity along with SAG-4 and MAG-1 bradyzoite genes is recommended for toxoplasmosis diagnosis using PBMC samples with real-time PCR.

## Data Availability

The data that support the findings of this study are available from the corresponding author upon reasonable request.

## Abbreviations

SAG-4: Surface antigen glycoprotein-4
MAG-1: Matrix antigen-1
PBMC: Peripheral blood mononuclear cell
ELISA: Enzyme-linked immunosorbent assay
PCR: Polymerase chain reaction
RT-PCR: Real-time PCR
FRET: Fluorescence resonance energy transfer
